# Accumulation and Transmission of ‘*Candidatus* Liberibacter solanacearum’ Haplotypes by the Nymphs of Two Psyllid Vectors

**DOI:** 10.3390/insects14120956

**Published:** 2023-12-16

**Authors:** Junepyo Oh, Maria Azucena Mendoza Herrera, Brenda Leal-Galvan, Svetlana Kontsedalov, Murad Ghanim, Cecilia Tamborindeguy

**Affiliations:** 1 Department of Entomology, Texas A&M University, College Station, TX 77843, USA; ohjunepyo@tamu.edu (J.O.); azucena.mendoza@ag.tamu.edu (M.A.M.H.); brenda.leal@tamu.edu (B.L.-G.); 2 Department of Entomology, Agricultural Research Organization, The Volcani Center, HaMaccabim Road 68, P.O. Box 15159, Rishon LeZion 7505101, Israel; svetlanak@volcani.agri.gov.il

**Keywords:** ‘*Candidatus* Liberibacter solanacearum’, potato psyllid, carrot psyllid, zebra chip, transmission

## Abstract

**Simple Summary:**

Psyllids (Hemiptera: Psylloidea) are vectors of ‘*Candidatus* Liberibacter solanacearum’ (Lso), a destructive bacterial plant pathogen affecting various crops. With the aim of preventing disease transmission, many studies have investigated the immune response that psyllids exhibit when acquiring Lso, in particular by adults. However, there are fewer data on acquisition and transmission by nymphs. In this study, we quantified the titer of two Lso haplotypes, LsoA and LsoB, in the gut of different instars of psyllid nymphs; assessed their transmission rates to tomato; and investigated the transmission of LsoD by carrot psyllid nymphs to celery. The results showed that LsoA reached higher titers in the gut of third-instar nymphs and was transmitted more efficiently by nymphs than LsoB. The transmission of LsoD increased as nymphs developed. Our findings provide fundamental knowledge of Lso transmission by nymphal stages for future consideration in management strategies.

**Abstract:**

‘*Candidatus* Liberibacter solanacearum’ (Lso) is a plant pathogenic bacterium transmitted by psyllids that causes significant agricultural damage. Several Lso haplotypes have been reported. Among them, LsoA and LsoB are transmitted by the potato psyllid *Bactericera cockerelli* and infect solanaceous crops, and LsoD is transmitted by the carrot psyllid *B. trigonica* and infects apiaceous crops. Several studies evaluated the transmission of these haplotypes by adult psyllids. However, fewer data are available on the transmission of different Lso haplotypes by psyllid nymphs. In this study, we investigated the transmission of these three haplotypes by psyllid nymphs to expand our basic understanding of Lso transmission. Specifically, the objective was to determine if the haplotypes differed in their transmission rates by nymphs and if LsoA and LsoB accumulated at different rates in the guts of nymphs as it occurs in adults. First, we quantified LsoA and LsoB titers in the guts of third- and fifth-instar potato psyllid nymphs. We found similar LsoA titers in the two nymphal stages, while LsoB titer was lower in the gut of the third-instar nymphs compared to fifth-instar nymphs. Second, we assessed the transmission efficiency of LsoA and LsoB by third-instar nymphs to tomato plants, revealing that LsoA was transmitted earlier and with higher efficiency than LsoB. Finally, we examined the transmission of LsoD by carrot psyllid nymphs to celery plants and demonstrated an age-related difference in the transmission rate. These findings provide valuable insights into the transmission dynamics of different Lso haplotypes by nymphal vectors, shedding light on their epidemiology and interactions with their psyllid vectors.

## 1. Introduction

‘*Candidatus* Liberibacter solanacearum’ (Lso) is a Gram-negative, phloem-limited, unculturable plant pathogenic bacterium from the Alphaproteobacteria class [1,2]. Lso is classified into genetic groups based on three approaches: the single nucleotide polymorphism (SNP) genotyping of the 16s rRNA, 16S/23S ISR, and 50S rplJ and rplL ribosomal protein genes [3]; multilocus sequence typing markers (MLST) [4]; and simple sequence repeat (SSR) [5]. These groups are known as haplotypes, and several haplotypes have been found infecting different crops [6,7,8,9,10]. In North America, at least two Lso haplotypes, A and B, infect solanaceous crops, causing zebra chip in potato [1,11]. Zebra chip is a devastating disease that can reduce crop production by up to 85% in seriously impacted fields [12]. These two haplotypes are vectored by the potato psyllid (also known as the tomato psyllid), *Bactericera cockerelli* (Šulc) (Hemiptera: Triozidae) [13]. Lso C, D, and E infect apiaceous crops, such as carrot and celery, in different parts of Europe, Northern Africa and Israel [6,7,14,15,16]. LsoD is found in the Mediterranean region, including Israel, and is transmitted by the carrot psyllid*, Bactericera trigonica* (Hodkinson).

Lso is transmitted by psyllids in a circulative and propagative manner [17,18]. Once Lso is ingested and reaches the gut lumen, it crosses the gut membrane and replicates in the gut epithelial cells before crossing into the hemocoel. Then, Lso infects other organs such as the salivary glands [13,19,20,21]. Because the psyllid gut is the first organ infected by Lso after ingestion, its colonization might be an essential component of Lso acquisition and transmission [22]. Moreover, the gut can be a barrier to pathogen transmission as it plays a crucial role in insect immunity [23,24,25,26]. Therefore, studying the Lso–psyllid interaction in the gut can provide fundamental knowledge to understand Lso transmission.

Psyllids are hemipteran insects that go through three phases during their life cycle: egg, nymph, and adult. There are five nymphal instars, and all nymphal stages feed abundantly, except during molting [27,28]. The ability of insect vectors to acquire and transmit pathogens can vary depending on their life stage. For example, adult Asian citrus psyllids, *Diaphorina citri*, are efficient vectors of ‘*Candidatus* Liberibacter asiaticus’ (CLas) only if they acquired the pathogen as nymphs [29]. Potato psyllid adults can acquire and transmit Lso [21,30,31]. A previous study in our laboratory showed that the two Lso haplotypes, A and B, have distinct acquisition and transmission rates by adult potato psyllids: the LsoB population increased more rapidly in the psyllid gut than the LsoA population, and LsoB had a higher transmission rate than LsoA following a 7-day acquisition access period (AAP) [30]. There are fewer data are available on the acquisition and transmission of Lso by nymphs. It is known that nymphs can efficiently transmit Lso within a short inoculation period of 48 h as early as the third instar, although with lower efficiency than adults [32]. However, the previous studies only evaluated the ability of nymphs to transmit when they were co-infected with LsoA and LsoB. Likewise, no study has focused on the acquisition and transmission of LsoD by nymphs.

Evaluating the acquisition and transmission of different Lso haplotypes by different vector species and/or vector life stages can help us understand the molecular mechanisms involved in these processes, and identify key molecular players that can be targets to develop transmission-blocking strategies. The objective of this study was to investigate the transmission of LsoA, LsoB, and LsoD by psyllid nymphs. For this purpose, first, we quantified the titer of LsoA and LsoB by quantitative real-time PCR (qPCR) in the gut of third- and fifth-instar potato psyllid nymphs. Second, we determined and compared the transmission rate of Lso A and LsoB by potato psyllid nymphs to tomato. Third, we tested the transmission rate of LsoD by first- to fifth-instar carrot psyllid nymphs to celery. This research will pave the way to discover the molecular interactions that occur between nymphs and Lso, with the goal of developing novel techniques to disrupt Lso transmission at early stages.

## 2. Materials and Methods

### 2.1. Plants and Psyllid Colonies

All experiments involving LsoA and LsoB, potato psyllids and tomato plants were performed in the Department of Entomology at Texas A&M University in Texas, United States. *Solanum lycopersicum* L. ‘Moneymaker’ tomato seeds (Vicotry Seed Company., Irving, TX, USA) were planted in containers using Sun Gro Sunshine LP5 mix (Bellevue, WA, USA). The Miracle-Gro Water-Soluble Tomato Plant Food (18-18-21 NPK; Scotts Miracle-Gro Company, Marysville, OH, USA) was applied to the obtained plants twice weekly according to the label rate. Plants were kept throughout the experiments under a photoperiod of 16 h light and 8 h dark at room temperature (24 ± 2 °C).

Colonies of potato psyllids harboring LsoA or LsoB haplotypes were maintained on tomato plants in insect-proof cages (24 by 13.5 by 13.5 cm; BioQuip, Compton, CA, USA). All psyllid colonies were kept separate and maintained under a photoperiod of 16 h light and 8 h dark at room temperature (24 ± 2 °C). The presence of Lso and the haplotype in each colony were tested regularly by PCR using the LsoF/012 primers for Lso detection and the SSR1 primers for haplotype determination [5,33].

In Israel, carrot psyllids were collected from a carrot field in Saad, Israel, and were maintained for several generations in the laboratory located in the Department of Entomology, Volcani Institute. LsoD-infected (Lso+) and Lso-free (Lso-) celery (*Petroselinum crispum*) plants were maintained in separate cages under 14 h photoperiodic light, 25 ± 2 °C, and 60% humidity, and new infected plants were consciously prepared using insect-mediated transmission of the bacteria. Psyllids were periodically tested for Lso using PCR analyses (Primers: Omp_F/Omp_R) [34].

### 2.2. Midgut Dissections and LsoA and LsoB Quantification in Guts

Potato psyllid guts were dissected in 1x PBS under the Leica EZ4W0037 stereomicroscope (Leica Microsystems, Wetzlar, Germany). For the experiments, third- and fifth-instar nymphs from the LsoA- and LsoB-infected colonies were collected and dissected separately. There were three biological replicates for each instar and haplotype, each one had 30 dissected midguts.

DNA was extracted from pools of thirty guts using the DNeasy blood and tissue kit (Qiagen, Hilden, Germany). Each biological replicate was used as an individual template for qPCR analyses. Lso was quantified by qPCR using the LsoF and HLBR primers [33,35]. Psyllid 28s rDNA was amplified as an internal control [36]. The qPCR was carried out using the SYBR Green Supermix Kit with a QuantStudio 6 Flex Real-Time PCR System (Applied Biosystems, Foster City, CA, USA). Each technical replicate contained 30 ng of DNA, 0.25 µL of each primer at 250 nM, and 1X SYBR master mix. The total volume per reaction was adjusted to 10 µL with nuclease-free water. The qPCR program was set to 95 °C (2 min), 95 °C (5 s per cycle), and 60 °C (30 s) for 40 cycles. There were two technical replicates for each reaction and a negative control in each run.

Lso in psyllid guts was standardized by calculating ∆ Ct = (Ct of Lso gene) − (Ct of psyllid 28S rDNA) from three biological replicates. A standard curve was prepared for the quantification of Lso in psyllid guts using a plasmid containing the Lso 16S rDNA target, as described by [37].

### 2.3. LsoA and LsoB Transmission Experiments

**Figure 1:**
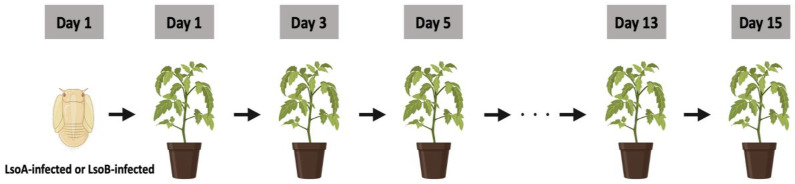
Sequential transmission test of third-instars potato psyllids harboring LsoA and LsoB. One nymph was transferred sequentially to a new non-infected tomato plant for a 2-day inoculation access period (IAP). Day 1 is the initial day. The experiment started with 16 nymphs from the LsoA-infected colony and 15 nymphs from the LsoB-infected colony being transferred to a different recipient non-infected tomato plant. After 4 weeks, the presence of Lso was tested in each plant (top-tier leaves) by PCR.

The sketch of the transmission assay is shown in (Figure 1). To determine the transmission efficiency of third-instar nymphs, a single third-instar nymph from the LsoA- or LsoB-infected colony was caged on a middle leaf of a tomato plant using a mesh bag. The nymph was allowed an initial inoculation access period (IAP) of 48 h. After the initial inoculation access period, the nymph was individually transferred using a standard paintbrush to a new tomato plant and allowed another 48 h IAP. The experiment was carried out until the psyllids became adults or died. There were at most 8 total transfers. For the LsoA transmission experiment, the experiment started with 16 nymphs, while the LsoB transmission experiment started with 15 nymphs. A set refers to the 15 plants or 16 plants per time point during the experiment. All plants were 4-5 weeks old when they were infested. Insect mortality and molting were recorded for each insect transfer. A group of nymphs was maintained in similar conditions for substitution in case nymphs died prematurely. The plants were maintained for at least four weeks after psyllids were removed and tested for infection as described below.

### 2.4. LsoD Transmission Experiments

Celery plants infected with LsoD were infested with Lso-free adult carrot psyllid females for a 26 h oviposition period inside a rearing box. The adult psyllids were then removed and the plants (source plants) with laid eggs were transferred to a new rearing box. Seven days after the oviposition, the eggs hatched. These age-synchronized first-instar nymphs were used for the transmission experiments. Every two days, a group of three or four nymphs was collected from the age-synchronized psyllid pool developing on the infected source plant and continuously acquiring the bacteria and transferred individually to a new uninfected celery plant (one insect per plant). Right after hatching, the first group of three nymphs was used for sequential transmissions. This setup created 8 groups of nymphs with the following ages: 0–1 days, 2–3, 4–5, 7–8, 9–10, 11–13, 14–15, and 16–17 days. A total of 30 individuals were used for the assays. The nymphs from each group were used in sequential transmission experiments by transferring them individually to a separate new plant every 2–3 days, as described for the potato psyllids (one insect per plant). The sequential transmission was continued until molting to adulthood. This allowed us to evaluate the transmission of individual nymphs starting at different ages. In total, there were 8 groups used. By the time the 8th group was collected, the psyllids were already 20 days old, and they were molting to adults. The plants were maintained for 2 weeks after psyllid removal and tested for infection as described below.

### 2.5. Lso Detection in Tomato and Celery Plants

To detect LsoA or LsoB, samples from the top-tier leaf were collected 4 weeks post-inoculation, and DNA was extracted, as previously described by [37]. The DNA was used to determine the presence of Lso with LsoF/012 primers, and each haplotype was verified using SSR1 primers [5,33]. The results were analyzed using gel electrophoresis.

To detect LsoD in celery plants, DNA was extracted from 100 mg leaf tissue by crushing them in liquid nitrogen and using the CTAB method [38]. The DNA was then purified using phenol-chloroform and was stored at −20 °C until further use. Real-time PCR analysis was carried out as described before by [34]. The CT values were normalized using primers for the glyceraldehyde-3-phosphate dehydrogenase (GAPDH) gene from celery [34].

### 2.6. Statistical Analysis

For the analysis of LsoA and LsoB genome copies in the gut of potato psyllid nymphs, a log 10-transformation was applied to the data. The log of Lso copies was taken to assure normality. The verification of homogeneity of variance was conducted using Levene’s Test, and the residuals were checked for normal distribution using the Shapiro–Wilk test. Data were analyzed using a two-way ANOVA, with Lso haplotype and nymphal stage as factors followed by Tukey’s post hoc test. For transmission analyses, a logistic regression model was fitted to examine the effects of two factors (two Lso haplotypes and the number of days post-acquisition) on the probability that a plant would become infected between 1 and 11 days, after which many of the psyllids became adults. The percentage of infected plants from the experiments at each time point was obtained using this model. The log-odds model is given by Log[p/(1 − p)] = b_0_ + b_1_ Haplotypes + b_2_ Days + b_3_Haplotypes∗Days + e, where p = the probability of a plant becoming infected after being exposed to the Lso haplotypes for a particular number of days, and p/(1 − p) is the odds of an exposed plant becoming infected [30]. The data were analyzed using JMP Version 16 (SAS Institute Inc., Cary, NC, USA), R (https://www.r-project.org/ accessed on 9 July 2023), and GraphPad Prism 9.5.1 Software (GraphPad Software, San Diego, CA, USA).

## 3. Results

### 3.1. LsoA and LsoB Titer in the Gut of Potato Psyllid Nymphs

**Figure 2:**
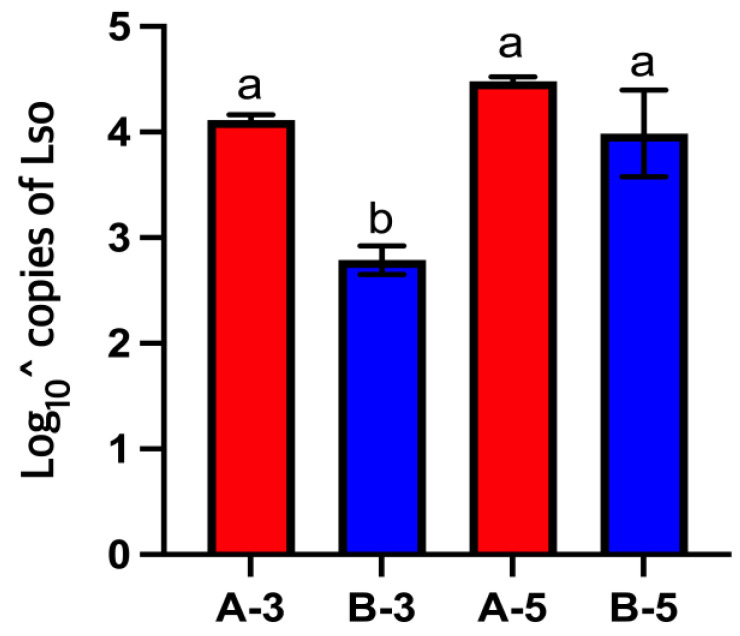
Bar graph representing the titer of LsoA and LsoB in the gut of third- and fifth-instar nymphs (*n* = 3). All values are represented as mean ± SD. The letters indicate statistically significant differences (*p* < 0.05). A-3 = third-instar of LsoA infected nymphs; B-3 = third-instar of LsoB infected nymphs; A-5 = fifth-instar of LsoA infected nymphs; B-5 = fifth-instar of LsoB infected nymphs.

Lso was quantified in the gut of third- and fifth-instar nymphs from the LsoA- and LsoB-infected colonies. A two-way ANOVA was performed to compare the Lso titer in the gut of third- and fifth-instar nymphs from the LsoA- and LsoB-infected colonies. There was a significant effect of the haplotype (F(1) = 52.0171, *p* < 0.001) and the instar (F(1) = 38.4955, *p* < 0.001) on the number of Lso copies in the psyllid gut. The interaction between these terms was also statistically significant (F(1, 8) = 10.89, *p* < 0.05). Third-instar nymphs from the LsoB-infected colony had a lower Lso titer in their guts than fifth-instar nymphs and nymphs from the LsoA-infected colony. No differences were measured among the fifth-instar nymphs from the LsoB-infected colony and the third- and fifth-instar nymphs from the LsoA-infected colony (Figure 2).

### 3.2. LsoA and LsoB Transmission by Potato Psyllid Nymphs

The sequential inoculation of tomato plants with a third-instar nymph was performed to verify if nymphs transmitted Lso haplotypes with the same efficiency. Nymphs from the LsoA-infected colony were able to transmit LsoA during the initial inoculation period of 48 h in the experiment (Table 1). This resulted in 50% of the plants being infected in the first set of plants. Overall, the transmission rate of LsoA-harboring nymphs was between 50% and 63% at each transfer, and it increased in the adult stage to 75% (Table 1).

In the experiment with nymphs from the LsoB-infected colony, the first LsoB-infected plants were detected after the third transfer. The transmission rate by nymphs was between 0% and 20% for each set of plants, but it increased in the adult stage, reaching as high as 33% (Table 2).

**Figure 3:**
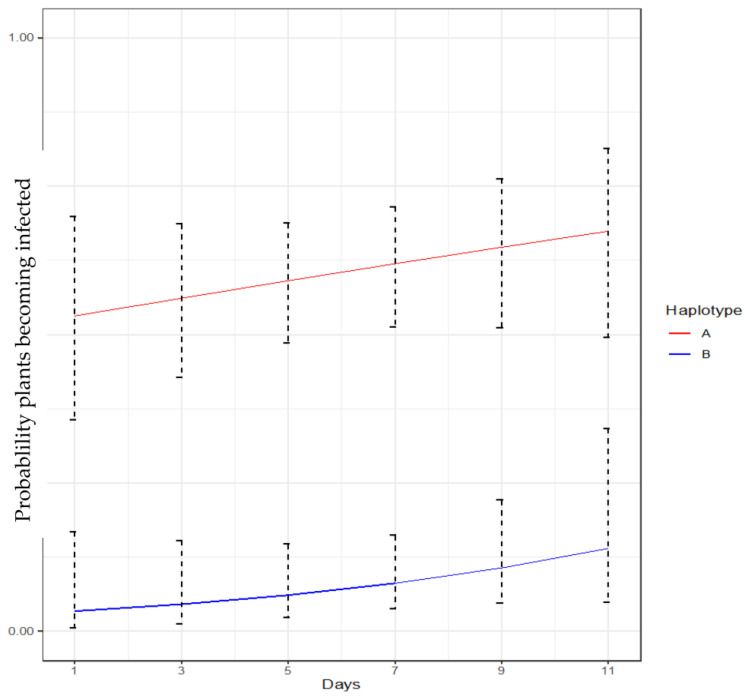
Sequential transmission test of third instars harboring LsoA or LsoB. Probability of plants being infected versus days post-acquisition with a 95% confidence interval (CI) based on the logistic regression model.

Based on the logistic regression model, there was a significant effect of the Lso haplotype (*p* < 0.01) but not of the number of days after removal post-acquisition (*p* = 0.32975) on the probability that a plant would become infected. Similarly, no significant effect of the haplotype-by-day interaction was observed (*p* = 0.50039), suggesting that the difference in infection probability between LsoA and LsoB remained comparatively the same throughout the experiment. In general, compared to LsoB, LsoA exhibited significantly higher odds (probability of infection) for the observed period (Figure 3).

### 3.3. LsoD Transmission by Carrot Psyllid Nymphs

As shown in Table 3, there were eight groups of nymphs with ages of 0–22 days used to investigate the LsoD transmission rates in carrot psyllid nymphs. The longer the psyllids remained on the source plant, the sooner after transfer they transmitted Lso for groups 1 and 5, corresponding to nymphs aged from 0–1 to 10–11 days old. The only exception was nymphs in group 4, which first transmitted LsoD after 9 days, while those in group 3 first transmitted LsoD after 7 days. Insects in the last group, group 8, were 16–17 days old insects and were already molting to adulthood; they did not transmit LsoD probably because they did not feed.

## 4. Discussion

‘*Candidatus* Liberibacter solanacearum’ (Lso) is a devastating bacterial plant pathogen. Currently, several haplotypes associated with different host plants and insect vector species have been reported [8,39]. Several studies have focused on the molecular interactions between the bacterium and the vector [26,40,41,42,43]. However, relatively few studies have focused on the transmission, acquisition, or accumulation of the different Lso haplotypes by their vectors [19,21,30,44]. Even fewer studies have focused on the Lso transmission by nymphs.

The present study was performed to compare the accumulation of LsoA and LsoB in the potato psyllid nymph gut and the transmission efficiency of these haplotypes by nymphs. It also aimed to report for the first time the transmission efficiency of LsoD by carrot psyllid nymphs. Comparing the acquisition and transmission of bacterial pathogens from different pathosystems can help us understand the epidemiology of diseases. This fundamental knowledge could shed light on common and unique features of their interaction with their vector. For instance, in the case of CLas, the Asian citrus psyllids must acquire the pathogen during the nymphal stages for efficient transmission during the adult stage [45], while potato psyllid adults can efficiently transmit Lso even if they acquired the pathogen as adults [30]. The potato psyllid can transmit Lso during the nymphal stage, as early as the third instar [32], but the studies were performed with LsoA and LsoB double-infected or LsoA-harboring psyllids [46,47].

In a previous study, we compared the accumulation of LsoA and LsoB in the gut of adults. We found that LsoA and LsoB titers accumulated to similar levels in the gut of adults, but LsoB reached the plateau six days earlier than LsoA. Further, we found a lower transmission efficiency for LsoA [30]. However, in the present study, we found that the LsoA titer had already reached a plateau in the third-instar nymphs, while the LsoB titer was lower in third-instar nymphs but increased in fifth-instar nymphs to similar levels to that of LsoA (Figure 2). This means that the plateau was reached sooner by LsoA than LsoB. Based on these results, we hypothesized that nymphs could transmit LsoA earlier than LsoB if reaching the plateau titer in the gut was a requirement for transmission. Alternatively, the nymphs could transmit LsoA more efficiently if the titer in the gut correlated with transmission efficiency. We found that the LsoA-infected colony’s nymphs could transmit the pathogen as third-instar nymphs; half of these nymphs infected the first set of plants, but the first transmission of LsoB did not occur until the third set of plants. These results indicated there was a lag in the transmission of LsoB compared to LsoA, and that the low amount of LsoB titer in the gut of third-instar nymphs could be a factor in the transmission lag.

We also observed differences in the transmission efficiency by nymphs: overall, LsoA was transmitted to 50–63% of the plants in each set (Table 1), yet the maximum transmission by nymphs for LsoB was 20% (Table 2). Moreover, based on the logistic regression model, LsoA had significantly higher odds (probability of infection) across 11 days relative to LsoB (Figure 3). The probability of LsoA detection did not increase during the assay, and transmission by older nymphs was as efficient as by third-instar nymphs. There was also an observed difference in the developmental time between the nymphs from the LsoA- and LsoB-infected colonies. The transmission experiments of these haplotypes were not run in parallel, so it was impossible to compare the development time of the nymphs. It is noteworthy that since we do not fully understand how each haplotype influences the development of the psyllid, it might be possible that the specific interaction influences the development time. This needs to be explored in more detail.

There are several factors causing differences in the accumulation and transmission between LsoA and LsoB in the nymphal stages. These differences could be due to the various immune responses of potato psyllids upon LsoA or LsoB infection. For instance, we previously identified differences in the expression of immune genes in the gut of adult psyllids in response to LsoA and LsoB infection [26,40]. Thus, the differences among the different Lso pathosystems could also be expected [25,40,41,42,43]. Indeed, differences in psyllid response to different Liberibacter pathogens have been reported. For example, while apoptosis was identified in the gut of adult Asian citrus psyllids infected with CLas [48], no evidence of apoptosis was uncovered in response to LsoA or LsoB in the gut of adult potato psyllids [42]. Furthermore, silencing an apoptosis-inhibitor gene decreased the acquisition of Lso and the transmission efficiency [25]. Nevertheless, similarities among the pathosystems also exist. For instance, autophagy was identified in response to LsoA, LsoB, and LsoD in the gut of the adult psyllids [40,41].

The bacterial pathogenicity could also affect its transmission. Indeed, we also reported differences in pathogenicity between LsoA and LsoB in potato psyllids; LsoB is more pathogenic than LsoA in potato psyllids [49]. Considering these different pathogenic characteristics, LsoA and LsoB might manipulate their vector differently, suggesting the possibility that the psyllid’s immune response to each haplotype might act differently. It is interesting that in the current experiments, only one nymph from the LsoA-infected colony died before reaching adulthood (two others were lost), while none of the nymphs from the LsoB-infected colony died because we have previously shown that Lso infection causes nymphal mortality, in particular LsoB [49]. It is also worth noting that no mortality was recorded among the carrot psyllid nymphs used in the LsoD transmission experiment.

Based on the obtained results, we speculate that LsoA is mainly transmitted by adults that acquired the pathogen as nymphs, while LsoB is transmitted by adults that acquired the pathogen as adults or nymphs. We cannot exclude the fact that allowing for LsoB acquisition by fourth- and fifth-instar nymphs could increase the transmission efficiency of this pathogen by older nymphs.

The experimental set-up to examine the transmission of LsoD allowed us to assess if the transmission was affected by the age of the nymph and/or the acquisition access period. The transmission rate of LsoD by nymphs increased during development. A higher percentage of transmission was observed when nymphs were allowed a longer LsoD acquisition access period (Table 3). Although we did not quantify the LsoD titer in the different nymphal stages, we speculate that the higher transmission rate could be a result of an increased amount of LsoD titer as the nymphs mature and acquire more Lso and/or the pathogen replicates in the nymphs.

Based on our results, LsoD could be transmitted by nymphs older than third instars (LsoD was not detected in the first set of plants for groups 4 or 5, which correspond to the third-instar nymphs), which is later than LsoA but earlier than LsoB. Further, the LsoD transmission rate was similar to or higher than that of LsoA. The differences in the transmission results of LsoA and LsoB compared to LsoD could also be the result of transovarial transmission as reported in [50] because LsoA- and LsoB-infected nymphs were collected directly from the infected colonies, whereas LsoD-infected nymphs were not. Based on our results, both potato and carrot psyllid nymphs can efficiently acquire and transmit LsoA and LsoD, whereas a longer acquisition access period might be needed for nymphs to efficiently transmit LsoB. Therefore, nymphs could contribute to the spread of the LsoA and LsoD to surrounding plants in fields. These differences in acquisition and transmission of LsoA and LsoB by nymphs combined with our earlier results studying the acquisition and transmission by adults [30] might explain some of the differences in the prevalence of the haplotypes observed in fields [51,52]. Future studies need to evaluate the accumulation of LsoD titer during the nymphal stages to determine if it increases throughout the nymphal development similar to LsoB, or if a plateau titer is reached at younger stages similar to LsoA. This information, coupled with gene expression analyses, can help us identify the different responses of psyllids to Liberibacter pathogens, and possibly identify pathways that could lead to reduced acquisition and transmission by nymphs and/or adults.

## 5. Conclusions

In this study, we compared the accumulation of LsoA and LsoB in third- and fifth-instar nymphs and the transmission efficiency of third-instar potato psyllid nymphs. We also reported for the first time the transmission efficiency of LsoD by carrot psyllid nymphal stages. The findings of our study indicate a higher accumulation and transmission efficiency of LsoA in third-instar potato psyllid nymphs than LsoB. In the case of LsoD, the transmission efficiency increased as the carrot psyllid nymphs matured and with longer acquisition access periods. These results contribute fundamental knowledge to understand and expand our current view of Lso transmission by psyllids, the vector capacity of nymphs and their role in the disease epidemiology. Future studies should compare the induction of different cellular and immune responses in response to the different haplotypes to identify specific responses that can delay the acquisition of the pathogen, its replication in the vector and its transmission to new plants.

## Figures and Tables

**Table 1:** LsoA transmission by potato psyllid nymphs.

LsoA	Days Post-Transfer							
Sample	1	3	5	7	9	11	13	15
1	+	+	−	−	−YA	+YA	+	−
2	−	+	−	+	+	+YA	+YA	+
3	+	+	+	−	+YA	+YA	+	−
4	+	+	+	+	+YA	+YA	+	+
5	−	+	+	+	−	+YA	+YA	+
6	+	−	+	−	−	+YA	+YA	+
7	−	−	+ (L/R)	+	+	−YA	+YA	+
8	−	+	−	+	−	−YA	−	+
9	+	+	−	+ (D/R)	+YA	+YA	+	−
10	−	−	−	+	−	−	+	+YA
11	−	+	+	−	+	+	+	−YA
12	−	−	−	+	+	+YA	+YA	+
13	−	+	+	−	+	−	+YA	+YA
14	+	+	+	+	+	−	−YA	+YA
15	+	−	−	+	+	+	+YA	+YA
16	+ (L/R)	−	+	−	−	+	−YA	+YA
Total %	50%	63%	56%	63%	63%	69%	81%	75%

Third-instar nymphs from the LsoA-infected colony were given sequential 48 h inoculation periods before being transferred to a new plant. The nymphs were followed into adulthood over a 15-day period. There was a total of 8 sets of plants with 16 plants each. The following symbols represent Lso-infected plants (+), uninfected plants (−), molting into young adults (YA), nymphs lost and replaced (L/R), nymphs dead and replaced (D/R). The last line shows the percentage of infected plants per set. Yellow color represents nymphs, green color represents young adults (light-colored teneral adults), and orange color represents mature adults (dark adults).

**Table 2:** LsoB transmission by potato psyllid nymphs.

LsoB	Days Post-Transfer							
Sample	1	3	5	7	9	11	13	15
1	−	−	−	−	+	−	−YA	−YA
2	−	−	−	−	−	−	−YA	−YA
3	−	−	−	−	−	−	+YA	+YA
4	−	−	+	−	−	+	−	+YA
5	−	−	+	+	−	−	−YA	−YA
6	−	−	−	−	−	−	−	−YA
7	−	−	−	−	−	−	−YA	−YA
8	−	−	+	−	−	−	−	−YA (L)
9	−	−	−	−	−	+	−YA	−YA
10	−	−	−	−	−	−	−YA	−YA
11	−	−	−	−	−	−	−YA	−YA
12	−	−	−	−	−	−	−YA	−YA
13	−	−	−	−	−	−	+YA	+YA
14	−	−	−	−	−	−	−	−YA
15	−	−	−	−	−	−	−	+YA
Total %	0%	0%	20%	7%	7%	13%	13%	33%

Third-instar nymphs from the LsoB-infected colony were given sequential 48 h inoculation periods before being transferred to a new plant. The nymphs were followed over a 15-day period until they reached adulthood. There was a total of 8 sets of plants with 15 plants each. The following symbols represent Lso-infected plants (+), uninfected plants (−), and molting into young adults (YA), and lost (L). The last line shows the percentage of infected plants per set. Yellow color represents nymphs, and green color represents young adults (light-colored teneral adults).

**Table 3:** LsoD transmission by carrot psyllid nymphs.

Plant	Group 1 0–1 Days (3)	Group 2 2–3 Days (3)	Group 3 4–5 Days (4)	Group 4 7–8 Days (4)	Group 5 9–10 Days (4)	Group 6 11–13 Days (4)	Group 7 14–15 Days (4)	Group 8 16–17 Days (4)
A—0 day	0%	0%	0%	0%	0%	0%	0%	0%
B—2 days	0%	0%	0%	0%	25%	0%	0%	0%
C—4 days	0%	0%	0%	0%	75%	0%	50%	**
D—7 days	0%	0%	50%	0%	75%	50%	**	
E—9 days	0%	33%	75%	75%	75%	25%		
F—11 days	0%	33%	75%	75%	**	**		
G—14 days	0%	33%	75%	**				
H—16 days	0%	33%	**					
I—18 days	33%	33%						
J—20 days	33%	**						
K—22 days	0%							

Newly hatched nymphs were maintained on the infected source plants. Every two days, a group of 3 or 4 nymphs was collected and placed individually in different plants. Each nymph was allowed a 2- or 3-day inoculation access period before being sequentially transferred to a new individual plant. There was a total of 30 nymphs assayed in 8 groups. For each group, the heading of the column reports the age of the nymphs in days and the number of individuals assayed in parenthesis. The first column reports the number of days since the group of nymphs was collected from the source plant. The table reports the percentage of plants that tested positive for LsoD for each group of nymphs per sequential transmission assay. ** represents adult emergence.

## Data Availability

All data that support the findings of this study are provided in the manuscript.

## References

[1] Munyaneza J., Crosslin J., Upton J. (2007). Association of *Bactericera cockerelli* (Homoptera: Psyllidae) with “zebra chip,” a new potato disease in southwestern United States and Mexico. J. Econ. Entomol..

[2] Jagoueix S., Bove J.-M., Garnier M. (1994). The phloem-limited bacterium of greening disease of citrus is a member of the α subdivision of the Proteobacteria. Int. J. Syst. Evol. Microbiol..

[3] Nelson W.R., Fisher T.W., Munyaneza J.E. (2011). Haplotypes of “*Candidatus* Liberibacter solanacearum” suggest long-standing separation. Eur. J. Plant Pathol..

[4] Glynn J., Islam M., Bai Y., Lan S., Wen A., Gudmestad N., Civerolo E., Lin H. (2012). Multilocus sequence typing of ‘*Candidatus* Liberibacter solanacearum’ isolates from North America and New Zealand. J. Plant Pathol..

[5] Lin H., Islam M.S., Bai Y., Wen A., Lan S., Gudmestad N.C., Civerolo E.L. (2012). Genetic diversity of ‘*Cadidatus* Liberibacter solanacearum’ strains in the United States and Mexico revealed by simple sequence repeat markers. Eur. J. Plant Pathol..

[6] Teresani G.R., Bertolini E., Alfaro-Fernández A., Martínez C., Tanaka F.A.O., Kitajima E.W., Roselló M., Sanjuan S., Ferrándiz J.C., López M.M. (2014). Association of ‘*Candidatus* Liberibacter solanacearum’ with a vegetative disorder of celery in Spain and development of a real-time PCR method for its detection. Phytopathology.

[7] Nelson W.R., Sengoda V.G., Alfaro-Fernandez A.O., Font M.I., Crosslin J.M., Munyaneza J.E. (2013). A new haplotype of “*Candidatus* Liberibacter solanacearum” identified in the Mediterranean region. Eur. J. Plant Pathol..

[8] Haapalainen M., Latvala S., Wickström A., Wang J., Pirhonen M., Nissinen A.I. (2020). A novel haplotype of ‘*Candidatus* Liberibacter solanacearum’ found in Apiaceae and Polygonaceae family plants. Eur. J. Plant Pathol..

[9] Haapalainen M., Wang J., Latvala S., Lehtonen M.T., Pirhonen M., Nissinen A. (2018). Genetic variation of ‘*Candidatus* Liberibacter solanacearum’ haplotype C and identification of a novel haplotype from Trioza urticae and stinging nettle. Phytopathology.

[10] Swisher Grimm K., Garczynski S. (2019). Identification of a new haplotype of ‘*Candidatus* Liberibacter solanacearum’ in *Solanum tuberosum*. Plant Dis..

[11] Munyaneza J.E., Goolsby J.A., Crosslin J.M., Upton J.E. (2007). Further evidence that zebra chip potato disease in the lower Rio Grande Valley of Texas is associated with *Bactericera cockerelli*. Subtrop. Plant Sci..

[12] Munyaneza J.E., Buchman J.L., Sengoda V.G., Fisher T.W., Pearson C.C. (2011). Susceptibility of selected potato varieties to zebra chip potato disease. Am. J. Potato Res..

[13] Mustafa T., Horton D.R., Cooper W.R., Swisher K.D., Zack R.S., Pappu H.R., Munyaneza J.E. (2015). Use of electrical penetration graph technology to examine transmission of ‘*Candidatus* Liberibacter solanacearum’ to potato by three haplotypes of potato psyllid (*Bactericera cockerelli*; Hemiptera: Triozidae). PLoS ONE.

[14] Mawassi M., Dror O., Bar-Joseph M., Piasezky A., Sjölund J., Levitzky N., Shoshana N., Meslenin L., Haviv S., Porat C. (2018). ‘*Candidatus* Liberibacter solanacearum’ is tightly associated with carrot yellows symptoms in Israel and transmitted by the prevalent psyllid vector *Bactericera trigonica*. Phytopathology.

[15] Munyaneza J., Fisher T., Sengoda V., Garczynski S., Nissinen A., Lemmetty A. (2010). First Report of “*Candidatus* Liberibacter solanacearum” Associated with Psyllid-Affected Carrots in Europe. Plant Dis..

[16] Tahzima R., Maes M., Achbani E., Swisher K., Munyaneza J., De Jonghe K. (2014). First report of ‘*Candidatus* Liberibacter solanacearum’ on carrot in Africa. Plant Dis..

[17] Cicero J.M., Fisher T.W., Qureshi J.A., Stansly P.A., Brown J.K. (2017). Colonization and intrusive invasion of potato psyllid by ‘*Candidatus* Liberibacter solanacearum’. Phytopathology.

[18] Cooper W.R., Sengoda V.G., Munyaneza J.E. (2014). Localization of ‘*Candidatus* Liberibacter solanacearum’ (Rhizobiales: Rhizobiaceae) in *Bactericera cockerelli* (Hemiptera: Triozidae). Ann. Entomol. Soc. Am..

[19] Vereijssen J., Smith G.R., Weintraub P.G. (2018). *Bactericera cockerelli* (Hemiptera: Triozidae) and *Candidatus* Liberibacter solanacearum in potatoes in New Zealand: Biology, transmission, and implications for management. J. Integr. Pest Manag..

[20] Cicero J.M. (2017). Stylet biogenesis in *Bactericera cockerelli* (Hemiptera: Triozidae). Arthropod Struct. Dev..

[21] Sengoda V.G., Cooper W.R., Swisher K.D., Henne D.C., Munyaneza J.E. (2014). Latent period and transmission of “*Candidatus* Liberibacter solanacearum” by the potato psyllid *Bactericera cockerelli* (Hemiptera: Triozidae). PLoS ONE.

[22] Fisher T.W., Vyas M., He R., Nelson W., Cicero J.M., Willer M., Kim R., Kramer R., May G.A., Crow J.A. (2014). Comparison of potato and Asian citrus psyllid adult and nymph transcriptomes identified vector transcripts with potential involvement in circulative, propagative Liberibacter transmission. Pathogens.

[23] Ohnishi J., Kitamura T., Terami F., Honda K.-i. (2009). A selective barrier in the midgut epithelial cell membrane of the nonvector whitefly *Trialeurodes vaporariorum* to Tomato yellow leaf curl virus uptake. J. Gen. Plant Pathol..

[24] Jassar O., Ghanim M. (2023). Association of endoplasmic reticulum associated degradation (ERAD) with the transmission of Liberibacter solanacearum by its psyllid vector. Insect Mol. Biol..

[25] Tang X.-T., Fortuna K., Mendoza Herrera A., Tamborindeguy C. (2020). Liberibacter, a preemptive bacterium: Apoptotic response repression in the host gut at the early infection to facilitate its acquisition and transmission. Front. Microbiol..

[26] Tang X.-T., Levy J., Tamborindeguy C. (2023). Potato psyllids mount distinct gut responses against two different ‘*Candidatus* Liberibacter solanacearum’ haplotypes. PLoS ONE.

[27] Abdullah N. (2008). Life history of the potato psyllid *Bactericera cockerelli* (Homoptera: Psyllidae) in controlled environment agriculture in Arizona. Afr. J. Agric. Res..

[28] Butler C.D., Trumble J.T. (2012). The potato psyllid, Bactericera cockerelli (Sulc) (Hemiptera: Triozidae): Life history, relationship to plant diseases, and management strategies. Terr. Arthropod Rev..

[29] Inoue H., Ohnishi J., Ito T., Tomimura K., Miyata S., Iwanami T., Ashihara W. (2009). Enhanced proliferation and efficient transmission of *Candidatus* Liberibacter asiaticus by adult *Diaphorina citri* after acquisition feeding in the nymphal stage. Ann. Appl. Biol..

[30] Tang X.-T., Longnecker M., Tamborindeguy C. (2020). Acquisition and transmission of two ‘*Candidatus* Liberibacter solanacearum’ haplotypes by the tomato psyllid *Bactericera cockerelli*. Sci. Rep..

[31] Sengoda V.G., Buchman J.L., Henne D.C., Pappu H.R., Munyaneza J.E. (2013). “*Candidatus* Liberibacter solanacearum” titer over time in *Bactericera cockerelli* (Hemiptera: Triozidae) after acquisition from infected potato and tomato plants. J. Econ. Entomol..

[32] Buchman J.L., Sengoda V.G., Munyaneza J.E. (2011). Vector transmission efficiency of liberibacter by *Bactericera cockerelli* (Hemiptera: Triozidae) in zebra chip potato disease: Effects of psyllid life stage and inoculation access period. J. Econ. Entomol..

[33] Li W., Abad J.A., French-Monar R.D., Rascoe J., Wen A., Gudmestad N.C., Secor G.A., Lee M., Duan Y., Levy L. (2009). Multiplex real-time PCR for detection, identification and quantification of ‘*Candidatus* Liberibacter solanacearum’in potato plants with zebra chip. J. Microbiol. Methods.

[34] Sarkar P., Kontsedalov S., Lebedev G., Ghanim M. (2021). The actin cytoskeleton mediates transmission of “*Candidatus* Liberibacter solanacearum” by the carrot psyllid. Appl. Environ. Microbiol..

[35] Li W., Hartung J.S., Levy L. (2006). Quantitative real-time PCR for detection and identification of *Candidatus* Liberibacter species associated with citrus huanglongbing. J. Microbiol. Methods.

[36] Nachappa P., Levy J., Pierson E., Tamborindeguy C. (2014). Correlation between “Candidatus Liberibacter solanacearum” infection levels and fecundity in its psyllid vector. J. Invertebr. Pathol..

[37] Levy J., Ravindran A., Gross D., Tamborindeguy C., Pierson E. (2011). Translocation of ‘*Candidatus* Liberibacter solanacearum’, the zebra chip pathogen, in potato and tomato. Phytopathology.

[38] Munyaneza J.E., Mustafa T., Fisher T.W., Sengoda V.G., Horton D.R. (2016). Assessing the likelihood of transmission of *Candidatus* Liberibacter solanacearum to carrot by potato psyllid, *Bactericera cockerelli* (Hemiptera: Triozidae). PLoS ONE.

[39] Grimm K.D.S., Horton D.R., Lewis T.M., Garczynski S.F., Jensen A.S., Charlton B.A. (2022). Identification of three new ‘*Candidatus* Liberibacter solanacearum’ haplotypes in four psyllid species (Hemiptera: Psylloidea). Sci. Rep..

[40] Tang X.-T., Tamborindeguy C. (2021). Identification of Autophagy-Related Genes in the Potato Psyllid, *Bactericera cockerelli* and Their Expression Profile in Response to ‘*Candidatus* Liberibacter Solanacearum’ in the Gut. Insects.

[41] Sarkar P., Ghanim M. (2022). Interaction of Liberibacter solanacearum with host psyllid vitellogenin and its association with autophagy. Microbiol. Spectr..

[42] Tang X.-T., Tamborindeguy C. (2019). No evidence of apoptotic response of the potato psyllid *Bactericera cockerelli* to “*Candidatus* Liberibacter solanacearum” at the gut interface. Infect. Immun..

[43] Oh J., Tamborindeguy C. (2023). Treatment of Rapamycin and Evaluation of an Autophagic Response in the Gut of *Bactericera cockerelli* (Sulč). Insects.

[44] Rashed A., Nash T., Paetzold L., Workneh F., Rush C. (2012). Transmission efficiency of ‘*Candidatus* Liberibacter solanacearum’ and potato zebra chip disease progress in relation to pathogen titer, vector numbers, and feeding sites. Phytopathology.

[45] Ammar E.-D., Ramos J.E., Hall D.G., Dawson W.O., Shatters Jr R.G. (2016). Acquisition, replication and inoculation of *Candidatus* Liberibacter asiaticus following various acquisition periods on huanglongbing-infected citrus by nymphs and adults of the Asian citrus psyllid. PLoS ONE.

[46] Sandanayaka W., Moreno A., Tooman L., Page-Weir N., Fereres A. (2014). Stylet penetration activities linked to the acquisition and inoculation of *C andidatus* L iberibacter solanacearum by its vector tomato potato psyllid. Entomol. Exp. Appl..

[47] Sandanayaka W., Tooman L., Hewett R. (2013). The impact of post acquisition period on detection of *Candidatus* Liberibacter solanacearum in tomato potato psyllid. N. Z. Plant Prot..

[48] Ghanim M., Fattah-Hosseini S., Levy A., Cilia M. (2016). Morphological abnormalities and cell death in the Asian citrus psyllid (*Diaphorina citri*) midgut associated with *Candidatus* Liberibacter asiaticus. Sci. Rep..

[49] Yao J., Saenkham P., Levy J., Ibanez F., Noroy C., Mendoza A., Huot O., Meyer D.F., Tamborindeguy C. (2016). Interactions “*Candidatus* Liberibacter solanacearum”—*Bactericera cockerelli*: Haplotype effect on vector fitness and gene expression analyses. Front. Cell. Infect. Microbiol..

[50] Hansen A., Trumble J., Stouthamer R., Paine T. (2008). A new huanglongbing species,“Candidatus Liberibacter psyllaurous,” found to infect tomato and potato, is vectored by the psyllid Bactericera cockerelli (Sulc). Appl. Environ. Microbiol..

[51] Dahan J., Wenninger E.J., Thompson B.D., Eid S., Olsen N., Karasev A.V. (2019). Prevalence of ‘*Candidatus* Liberibacter solanacearum’ haplotypes in potato tubers and psyllid vectors in Idaho from 2012 to 2018. Plant Dis..

[52] Wen A., Johnson C., Gudmestad N.C. (2013). Development of a PCR assay for the rapid detection and differentiation of ‘*Candidatus* Liberibacter solanacearum’ haplotypes and their spatiotemporal distribution in the United States. Am. J. Potato Res..

